# Connecticut Valley Dental Society

**Published:** 1887-11

**Authors:** Geo. A. Maxfield

**Affiliations:** Secretary


### CONNECTICUT VALLEY DENTAL SOCIETY.

SEMI-ANNUAL MEETING, HELD IN MONTREAL, P. Q., JULY 19 TO 21, INCLUSIVE.

REPORTED BY GEO. A. MAXFIELD, D. D. S., SECRETARY.

CONTINUED FROM PAGE 541.

Prof. Chas. Mayr read a paper on "General Health, as shown by the work of the Kidneys in relation to Teeth" (see page 578).

*Dr. Fones*—It has been stated that blood poisoning often occurs after the system has been overtaxed.

*Prof. Mayr*—Blood poisoning may occur without a previous overtaxed system. Excretions of lime-salts occur as soon as exertion commences. Urea is produced by regular work, but far more by worry. There is some doubt as to the source of uric acid. Muscular labor produces urea, yet the creatine in muscle does not seem to be changed. I do not doubt that we have produced, besides urea, something that has not been isolated. The best remedy to cleanse the system is to use waters that are known for their purity. Drink copiously of distilled water sufficient to flush the system. Patients frequently come having a foul breath. When this is not caused by the teeth, it is an indication that the system is in a disturbed condition. It shows that we ought to endeavor to strengthen the system by giving pure water. Blood-purifying remedies are useless ; there is nothing so good as pure water.

*Dr. Black*—We are continually liable to run to extremes in this matter. Bad breath may be caused by micro-organisms, but I have had patients with foul breath where I would not find micro-organisms. We are not sure that we have rendered the breath sweet when we have made the mouth pure.

*Prof. Mills*—I have been very much interested in the reading of this paper. This branch of the medical profession is evidently teaching the other branches. In every organ there is a power to do extra work. There is also a relation between all the excretions of the body, and especially between the urine and the fæces, and a person's breath is affected through the mechanical transfer from the alimentary canal. The membranes of the throat and mouth produce certain excretions which contaminate the breath. Then there is a relation between constipation and a relaxed throat. In these cases good results will follow flushing the system with pure water.

*Dr. Atkinson*—I am profoundly impressed at seeing a dental body listening to foundation principles. Our generalizations must stop until we hear more from the specialist. This is a very important subject, and we should continue investigations in this line.

Subject passed.

